# Emerging Trends and Challenges of IoT in Smart Healthcare Systems, Smart Cities and Education

**DOI:** 10.3390/s24175735

**Published:** 2024-09-04

**Authors:** Faheem Khan

**Affiliations:** Department of Computer Engineering, Gachon University, Seongnam-si 13120, Republic of Korea; faheem@gachon.ac.kr

Due to the rapid growth of science and technology, many modern devices are being developed to support healthcare and education systems. Modern hospitals and education systems face a number of challenges when treating patients. The current progress in the healthcare domain aims to support the complete monitoring of patients during the various stages of a treatment process. Similarly, the COVID-19 pandemic changed the shape of education systems for students, faculty and parents.

Currently, the Internet of Things (IoT) is gaining popularity, with an enormous effect on virtually everything and the capacity to remodel the digital world by connecting everything to the internet. There are a few IoT applications relevant to smart industries, smart cities, healthcare systems, education, etc. The IoT framework is complex and heterogeneous and brings a range of challenges, including decentralization, poor interoperability, privacy concerns and vulnerability to attacks. Because of its many advantages, such as distributed data storage and immutability, blockchain has become the solution for IoT safety, with the potential to improve the overall safety of the IoT ecosystem.

This Special Issue is focused on 13 selected topics from different fields related to healthcare systems, smart cities, industries and educational institutions using IoT to share novel research findings concerning IoT convergence, ranging from overviews to evidence-of-concept case studies to applications.

The Internet of Things (IoT) is an interconnection of devices that saves and collects data from neighboring devices or environments and can be termed an ecosystem of interconnected devices. In the 21st century, IoT is considered very important in the digital infrastructure for the remote regulation of digital devices. IoT facilitates the use of digital devices to connect society, business, education and healthcare. Such devices range from nanochips to routers, along with actuators, sensors and other related devices to connect with each other [1].

After COVID-19, IoT is considered a backbone for the healthcare system as well as for other institutions like the education system. In COVID-19, infected cases totaled 704,753,890, deaths totaled 7,010,681 and recovered cases totaled 675,619,811 until 13 April 2024. These numbers show the seriousness of the situation, and researchers worked hard to prevent the spread of the disease along with prevention and care. Researchers have found that in such pandemics, IoT is the best solution to communicate about the awareness, spread and precautions related to a disease by obtaining data through state-of-the-art devices with less human involvement [2].

IoT in healthcare, along with blockchain, is the most important and has many advantages, including improved security, decentralization and privacy. Implementing blockchain technology in IOT in healthcare in situations such as COVID could provide a feasible solution in terms of integration, privacy and interoperability. Due to its inherent features like smart contracts, hashed link models, encryption, etc., it enables healthcare systems to store medical records [3].

Many fields related to COVID-19 data analysis, like AI along with IoT, blockchain, machine learning and deep learning, collectively combatted COVID-19. During COVID-19, IoT was used to detect and forecast about patients remotely and helped avoid fake news and conspiracy theories about the spread of the disease and people’s deaths on social media. However, through blockchain along with AI, such conditions as explained can be avoided through the privacy and security provided by blockchain technology [4]. 

For COVID diagnosis, scientists and researchers performed a reverse transcription polymerase chain reaction, which is costly in terms of money and time. For COVID-19 diagnosis, two tests were repeatedly performed, i.e., computed tomography and chest radiography [5]. Nowadays, experienced and highly paid radiologists are required for the detection of chest-related diseases using X-rays because it is very difficult to detect chest disease through traditional X-rays due to their high error rate. Training a radiologist in a pandemic situation will be time consuming and expensive, and not good for the patients. Therefore, a computer-aided system is needed to be able to easily detect the virus and to save time and money. Computer-aided systems require privacy and security, which are the major concerns; therefore, researchers are focusing on introducing blockchain technology [6].

Cybersecurity is another important field in order to secure data, businesses, personal information, property, industrial information, etc. from attackers. However, the need for cybersecurity is very demanding in situations such as COVID-19. Researchers and scientists are working to prepare best practices for protecting computers and devices from cyber threats [7].

The COVID-19 era has produced many challenges for business, health care and education systems as most of employees worked online from home. Therefore, different institutions and businesses shifted towards digitization, where the main concern was cybersecurity. The increase in work from home during COVID-19 further attracted researchers to the significant cybersecurity threats. During COVID-19 47% of employees or users faced cybersecurity threats in the form of phishing scams. This paper [8,9] reviewed the increase in cyberattacks during the pandemic. It highlighted different cyberattacks on different organizations. Most of the cyberattacks were executed on employees through social engineering by tricking them into opening suspicious links sent through social media platforms. 

After COVID-19, countries executed different safety measures for employees to join the office, like maximizing ventilation. Monitoring indoor carbon dioxide was an important preventive strategy during COVID-19, and we are proposing to keep the level of carbon dioxide below 900 ppm. This threshold will ensure the safety and security of people during pandemics [10,11,12,13,14,15,16,17,18,19,20]. 

This Special Issue hopes to offer a forum for researchers and industry professionals to share novel research findings concerning IoT convergence, ranging from overviews to evidence-of-concept case studies to applications. In this Special Issue, 49 papers were submitted, and only 13 quality papers were selected for this Special Issue, which are listed below.

The paper by Al-Kahtani et al. discusses the challenges of different technologies and their applications relevant to the healthcare system. The paper searched different databases for the collection of relevant data and found that using IoT with different technologies will allow IoT to be accessible, cheap and pervasive anytime and anywhere with computational improvement. At the end of the paper, it is concluded that IoT along with smart technology can be very useful in pandemic situations like COVID-19 and can help the healthcare system save lives in critical situations.

The paper by Al-Atawi et al. discusses the importance of IoT in detail, especially the use of IoT in improving healthcare performance in COVID-19. In the paper, it is discussed that frequent visits of patients during COVID-19 make the situation worse in terms of virus spread, cost and death rate. In this paper, it is explained that by using IoT, the healthcare system can easily treat a patient without spreading the disease and without frequent visits to the doctor, resulting in lower costs and a lower death rate. Furthermore, the paper identifies that by using IoT, the symptoms of the patient can be identified, as can the patient’s location during COVID-19. 

Muhammad Junaid et al. explained in detail about analyses and their effect on human behavior. Information and data relevant to COVID-19 are very important and can assist institutions in the prevention of disease and its spread. This paper discusses how the integration of machine learning, deep learning, AI and IoT can help society and government to fight against COVID-19 as well as diagnose the patient. This paper also discusses the spread of fake news through social media. Finally, different analyses and techniques offer some future directions and guidelines for the government and society.

Asma Kanwal et al. explain the development of a cognitive architectural framework for self-aware and conscious agents. The nature-inspired humanoid cognitive computing platform for self-aware and conscious agents (NiHA) consists of cognitive applications, theories, a machine consciousness (MC) framework and artificial intelligence. The paper concentrates on mechanisms for service agents towards MC. This paper also discusses the social influence of psychological conditions. In the proposed paper, the agent has 90% accuracy in attention generation and 89% in terms of contextual-based working by examining psychological conditions through parallel selective attention.

Muhammad Tahir Naseem et al. suggested a methodology for COVID-19 detection relevant to chest disease. Chest disease is detected through an X-ray image by applying Transfer Learning algorithms. In this paper, two datasets, i.e., Dataset-1 and Dataset-2 were formed from a public database. In Dataset-1, 6000 X-ray images are collected within four classes, and in Dataset-2, 7200 images were collected consisting of six classes. For model training and testing, TL, with nine pretrained CNNs was implemented with preprocessing and augmentation methods. 

Mohammad Hijji et al. and Li Pingping et al. explains that cloud computing companies use huge data centers, consisting of virtual computers that are positioned worldwide and necessitate exceptionally high-power costs. The increased requirement for energy consumption in IT firms has posed many challenges for cloud computing companies pertinent to power expenses. Energy utilization is reliant upon numerous aspects, for example, the service level agreement, techniques for choosing the virtual machine, the applied optimization strategies and policies, and kinds of workloads. The present paper tries to provide an answer to challenges related to saving energy through the assistance of both dynamic voltage and frequency scaling techniques for gaming data centers. It also evaluated dynamic voltage and frequency scaling techniques compared to non-power-aware and static threshold detection techniques. The findings will help service suppliers face the quality of service and experience limitations by fulfilling service level agreements. For this purpose, the CloudSim platform is applied to the application of a situation in which game traces are employed as a workload for analyzing the procedure. The findings evidenced that an assortment of good quality techniques can help gaming servers conserve energy and sustain the best quality of service for consumers located throughout the world. The originality of this research presents a prospect to examine which procedure performs well (for example, dynamic, static, or non-power aware). The findings validate that less energy is utilized by applying a dynamic voltage and frequency method, along with fewer service level agreement violations and better quality of service and experience, in contrast with static threshold consolidation or the non-power-aware technique.

This Special Issue discusses emerging trends and challenges of IoT in smart healthcare systems, smart cities, education and different industries. Most of the data are collected either through ordinary literature reviews or systematic literature reviews [13], which will guide academia, researchers or institutions in the future pandemics. It will also prepare academia, researchers or institutions for the preparations that they should make.
